# Tailored therapeutic decision of rheumatoid arthritis using proteomic strategies: how to start and when to stop?

**DOI:** 10.1186/s12014-023-09411-2

**Published:** 2023-06-10

**Authors:** Shuo-Fu Chen, Fu-Chiang Yeh, Ching-Yun Chen, Hui-Yin Chang

**Affiliations:** 1 Department of Heavy Particles & Radiation Oncology, Taipei Veterans General Hospital, Taipei, Taiwan; 2 Division of Rheumatology, Immunology and Allergy, Department of Internal Medicine, Tri-Service General Hospital, National Defense Medical Center, Taipei, Taiwan; 3 Department of Biomedical Sciences and Engineering, Institute of Biomedical Engineering and Nanomedicine, National Central University, Taoyuan, Taiwan; 4 Institute of Biomedical Engineering and Nanomedicine, National Health Research Institutes, Miaoli, Taiwan; 5 Department of Biomedical Sciences and Engineering, Institute of Systems Biology and Bioinformatics, National Central University, No. 300, Zhongda Rd., Zhongli District, Taoyuan, 320317 Taiwan

**Keywords:** Rheumatoid arthritis, Proteomics, Biomarkers, Prediction, Treatment response

## Abstract

Unpredictable treatment responses have been an obstacle for the successful management of rheumatoid arthritis. Although numerous serum proteins have been proposed, there is a lack of integrative survey to compare their relevance in predicting treatment outcomes in rheumatoid arthritis. Also, little is known about their applications in various treatment stages, such as dose modification, drug switching or withdrawal. Here we present an in-depth exploration of the potential usefulness of serum proteins in clinical decision-making and unveil the spectrum of immunopathology underlying responders to different drugs. Patients with robust autoimmunity and inflammation are more responsive to biological treatments and prone to relapse during treatment de-escalation. Moreover, the concentration changes of serum proteins at the beginning of the treatments possibly assist early recognition of treatment responders. With a better understanding of the relationship between the serum proteome and treatment responses, personalized medicine in rheumatoid arthritis will be more achievable in the near future.

**Supplementary Information:**

The online version contains supplementary material available at 10.1186/s12014-023-09411-2.

## Introduction

Rheumatoid arthritis (RA) is a highly heterogeneous autoimmune disease characterized by chronic inflammation and joint destruction. Recent advances in biological disease-modifying anti-rheumatic drugs (bDMARDs) have revolutionized the management of RA [1], with four main groups available: anti-CD20 antibody rituximab, cytotoxic T lymphocyte-associated antigen 4‐immunoglobulin (CTLA4-Ig) abatacept, interleukin (IL)-6 receptor inhibitors (such as tocilizumab and sarilumab), and tumor necrosis factor inhibitors (TNFi) which include infliximab, adalimumab, golimumab, certolizumab, and etanercept. Furthermore, targeted small molecule inhibitors (e.g., tofacitinib and baricitinib) have emerged as new therapeutic options. Nevertheless, timely selections of appropriate treatments for individual RA patients remains challenging. Biomarkers for the characterization of different RA phenotypes are urgently needed to develop personalized treatment plans.

Proteomics is a valuable research for identifying functional molecules directly involved in the pathophysiology of RA. Serum protein analysis has been widely adopted in clinical practice for its less invasive nature and easy reproducibility compared with synovial sampling [2]. Mass spectrometry (MS) and immunoassays are two conventional methodologies for serum protein analysis. The former is a high-throughput technique to identify and quantify proteins by measuring their mass-to-charge ratios (m/z) and signal intensities. Proteins are ionized by electron ionization, electrospray ionization, or matrix-assisted laser desorption/ionization, and then separated based on mass-to-charge ratios before detection. MS is a powerful tool for identifying unknown compounds, determining the purity of a sample, and analyzing complex mixtures. On the other hand, immunoassays are used to detect and quantify specific proteins in a sample based on the specific binding between an antibody and its corresponding target molecules. In a typical immunoassay, a specific antibody is coated on a plate, and the sample is added to the plate. If the target molecule is present in the sample, it will bind to the antibody, forming an antigen–antibody complex that can be detected using a secondary antibody conjugated with an enzyme or fluorophore. The intensity of the signal generated by the enzyme or fluorophore is proportional to the amount of the target molecule in the sample, allowing for quantification. Both MS and immunoassays have their own unique strengths and limitations, and the choice of techniques mostly depends on specific experiment goals and sample characteristics [3].

**Fig. 1:**
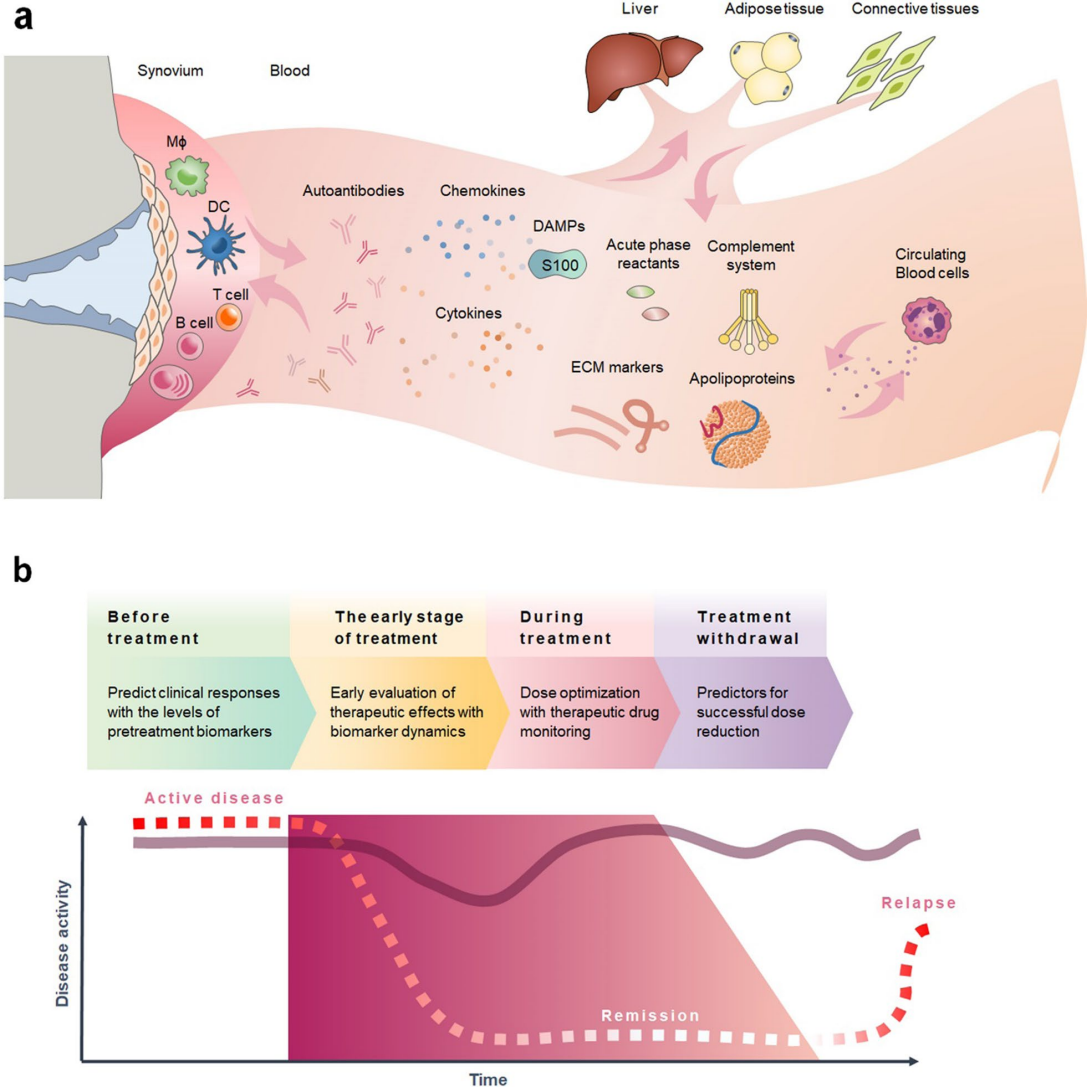
Serum proteome in the therapeutic decision of rheumatoid arthritis.** a** The serum proteome results from the interaction between inflamed joints and various tissues, such as liver, adipose tissue, connective tissues, and circulating blood cells. *Mɸ* macrophages, *DCs* dendritic cells, *DAMPs* danger-associated molecular pattern molecules, *ECM* extracellular matrix. **b** Serum proteins can aid in clinical decision-making in different stages of RA treatments. The trapezoid shadow represents the period of treatment administration followed by gradual tapering. The dashed line depicts the changes in disease activity in patients who achieved remission with treatments but relapsed after treatment withdrawal. The solid line illustrates the changes in disease activity in non-responders

In RA patients, serum proteins derived from the inflamed joints can exert systemic effects through interaction with liver, adipose tissue, connective tissues and circulating blood cells (Fig. 1a). Understanding the serum proteome has the potential to uncover fundamental factors that drive the immunopathogenesis of RA, thereby improving its management. Despite growing interests in serum proteomics, there is currently a lack of collective evidence to clarify their relevance in predicting treatment responses. To bridge this gap, our study aims to explore the clinical applications of serum proteins in four treatment stages of RA (Fig. 1b), including (1) *predicting clinical responses before treatments*. Since there is currently no effective cure, a trial-and-error process remains a common strategy for RA patients, which is very time-consuming and expensive. It is estimated that about one third of RA patients do not respond to the initial medications [4]. Bergman et al. have shown that predicting responses before treatment can offer several advantages for RA patients, such as improving outcomes, reducing costs, and minimizing exposure to ineffective medications [5]. Therefore, in the first stage, we will investigate the potential of serum proteins as indicators before the treatments. (2) *early evaluation of therapeutic effectiveness for treatment adjustments.* Most physicians rely on clinical experiences and patient-reported outcomes to evaluate treatment effectiveness, which could be subjective and may not truly reflect the underlying disease activity. In the second stage, we will discuss whether biomarker dynamics can be used as an objective measure of early response or non-response to facilitate treatment adjustments. (3) *therapeutic drug monitoring during treatments*. RA is a chronic disease that requires ongoing management, and the drug concentrations in serum can vary between individuals. Additionally, some RA medications can have potential side effects, so monitoring is important to ensure that the treatment is working well and not causing any harm. In the third stage, we will explore the utility of drug levels and anti-drug antibodies in guiding dose modification strategies. (4) *predicting successful treatment withdrawal after achieving clinical remission*. In some cases, it may be possible to withdraw or reduce the dose of RA medication if the disease is well-controlled and the patient has been in remission for a certain period of time. However, the timing and process of treatment withdrawal should be carefully managed to avoid disease flares or recurrence. Molecular remission, referring to a state in which the disease activity is undetectable at the molecular level, has increasingly become an important goal in the treatments of RA [6, 7]. In the fourth stage, we will investigate the relationship between the RA patients’ serum proteins and molecular remission to offer insights on successful treatment discontinuation. The possible bottlenecks occurred in each stage are summarized in Box 1.

To explore the clinical applications of serum proteins in RA treatments, a literature search was performed using PubMed, Embase, and Cochrane Library databases from four aspects: "the patient populations (rheumatoid arthritis)", " the purpose of the study (predict)", "serum protein biomarkers", and "outcome parameters". All keywords are listed in (Additional file 2: Table S1). As a result, 1553 articles were found, among which 476 were duplicates, and only the articles focused on serum protein biomarkers in the observational cohorts or clinical trials of RA patients were kept through manual inspection. Finally, a total of 276 articles were selected and discussed in the following paragraphs.

Box 1: The potential bottlenecks in the four RA treatment stages

1. Predicting clinical responses before treatments
  High variability in patient responses to bDMARDs.
  Lack of reliable biomarkers for predicting treatment responses.
  Failure to identify effective bDMARDs pose additional economic burdens and side effects.
  Limited understanding of the underlying pathogenesis and heterogeneity of RA.
2. Early evaluation of therapeutic effectiveness for treatment adjustments
  Lack of an objective early-stage assessment of therapeutic effectiveness.
  Difficulties in distinguishing early responses from transient fluctuations through clinical evaluation.
  Heterogeneity in the timing of early responses.
3. Therapeutic drug monitoring during treatments
  Variable pharmacokinetics of individual patients.
  Patient-reported outcomes may not reflect true disease activities.
  Lack of well-established associations between drug levels, anti-drug antibodies, and clinical responses.
  Uncertainty of the optimal frequency and time points for monitoring.
4. Predicting successful treatment withdrawal after achieving clinical remission
  Difficulties in distinguishing true remission from low disease activity or spontaneous fluctuations.
  Limited knowledge of the underlying mechanisms and biomarkers associated with successful withdrawal.
  High risk of disease relapse and joint damage if treatment is withdrawn prematurely.

### Predicting clinical responses before treatments

Predicting treatment responses remains challenging for RA patients. Serum proteins claimed to be useful for predicting therapeutic effectiveness in some studies may be considered irrelevant in other studies. Therefore, we will thoroughly discuss the relationship between serum proteins and the prediction of clinical responses in this section.

#### Autoantibodies

**Fig. 2:**
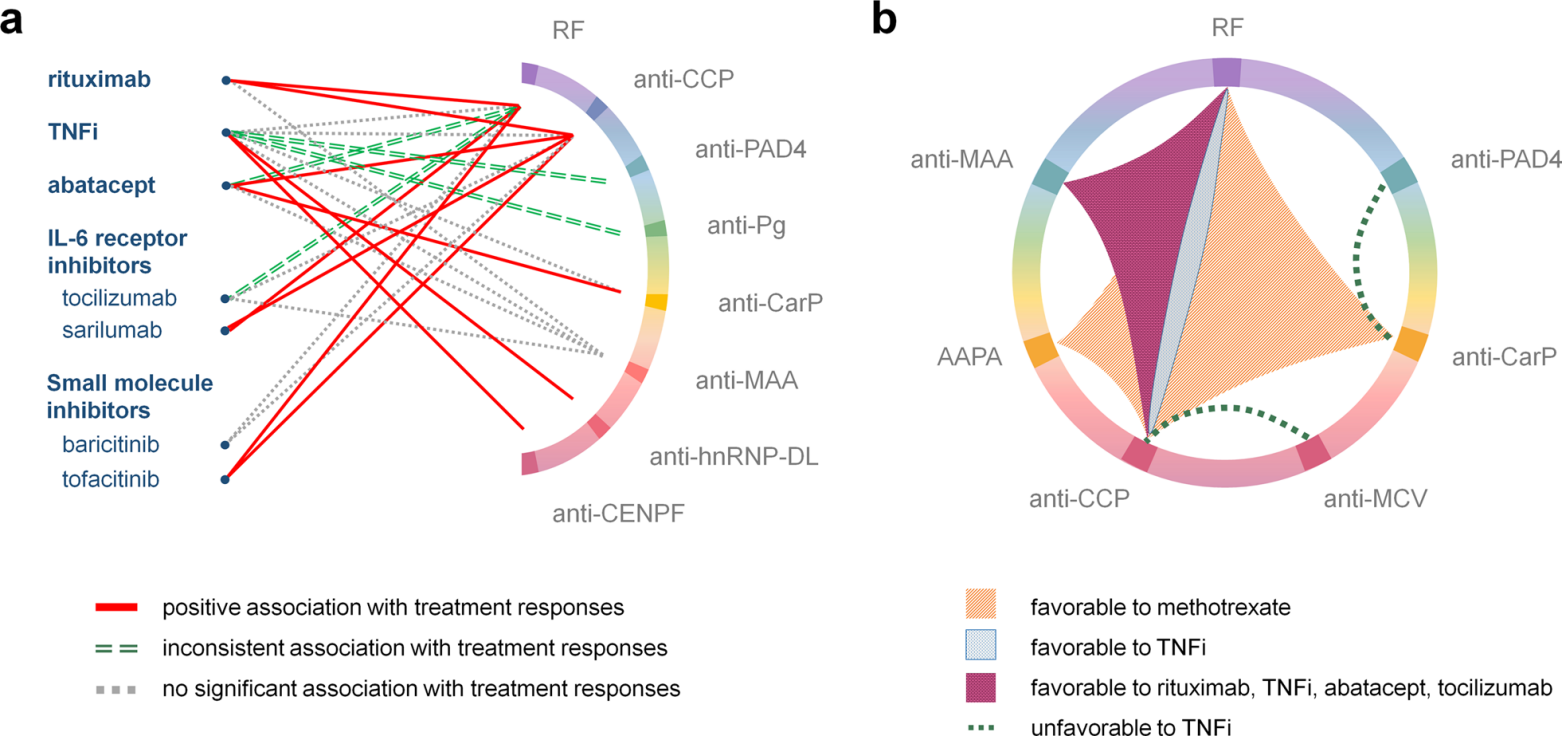
Autoantibodies in the prediction of treatment responses. **a** The relationship between the single positivity of antibodies and the treatment outcomes in RA patients, with red lines indicating a positive association, double dashed green lines indicating an inconsistent association, and dashed grey lines indicating no significant association. **b** The coexistence of antibodies associated with favorable treatment responses is illustrated by connected areas, while the combination of antibodies associated with unfavourable responses is depicted by dashed lines. The references are provided in Additional file 2: Table S2. *Anti-PAD4* anti-peptidylarginine deiminase 4, *anti-Pg* anti-Porphyromonas gingivalis, *anti-CarP* anti–carbamylated protein, *anti-MAA* anti-malondialdehyde-acetaldehyde adducts, *anti-hnRNP-DL* anti-heterogeneous nuclear ribonucleoproteins-D-like protein, *anti-CENPF* anti-centromere protein F, *anti-MCV* anti-mutated citrullinated vimentin, *AAPA* anti-acetylated peptide antibodies

A number of autoantibodies have shown promise in predicting treatment responses, as illustrated in Fig. 2a. Rheumatoid factors (RF) and antibodies against cyclic citrullinated peptide (anti‐CCP) are two autoantibodies that play pivotal roles in the diagnosis and classification of RA. RF is an antibody against the fragment crystallizable (Fc) region of immunoglobulin G (IgG) and anti‐CCP is a subset of anti-citrullinated protein antibodies (ACPAs) targeting citrullinated antigens. Based on our survey, both RF and anti-CCP are positively associated with treatment responses to rituximab, and are insignificantly associated with TNFi (Fig. 2a), indicating that RF and anti-CCP are potentially useful for the prediction of treatment responses to rituximab. This investigation is also consistent with a pooled analysis of 16 RA registries [8]. Interestingly, the relationships between autoantibodies and treatment responses are not always consistent for the drugs belonging to the same group. For example, both sarilumab and tocilizumab are IL-6 receptor inhibitors, and the presence of RF or anti-CCP is positively associated with the treatment responses to sarilumab but not to tocilizumab [9]. A similar phenomenon is observed in the small molecule inhibitors, where tofacitinib is positively associated with RF or anti-CCP while baricitinib is not [10, 11] (Fig. 2a).

Proteins participating in the process of citrullination, such as the enzymes peptidylarginine deiminase 4 (PAD4) and the periodontitis-causing bacteria Porphyromonas gingivalis (Pg), can also trigger the production of autoantibodies (anti-PAD4 and anti-Pg) in RA patients. Nevertheless, based on our investigation, it is still inconclusive to justify the use of anti-PAD4 and anti-Pg as the predictors of treatment responses in RA [12–17]. In addition to citrullination, there are several autoantibodies produced against other post-translational modifications (PTMs) of proteins, including carbamylation (non-enzymatic conversion of lysine to homocitrulline in the presence of cyanate), acetylation (enzymatic addition of an acetyl group) and lipid peroxidation-generated adducts, which may also be helpful for the prediction of treatment responses [18–20]. For example, the presence of anti–carbamylated protein (anti-CarP) antibodies are reported to be associated with better clinical improvement using abatacept [21], but the similar observation was not found in treatments with TNFi [12]. Another example is antibodies against malondialdehyde-acetaldehyde adducts (anti-MAA). MAA adducts generated through lipid peroxidation are overexpressed in a variety of conditions with oxidative stress. Although the circulating levels of anti-MAA correlate with the extent of tissue damage [22, 23], no significant association was found between anti-MAA status and treatment responses to TNFi, tocilizumab, abatacept, and rituximab [24, 25] (Fig. 2a).

Noteworthy, autoantibodies against nuclear antigen recently emerged as potential predictors for TNFi, including antibodies against heterogeneous nuclear ribonucleoproteins-D-like protein (anti-hnRNP-DL) and centromere protein F (anti-CENPF) [26, 27]. These antibodies may be elicited during externalization of intracellular neoepitopes via neutrophil extracellular traps. A prospective investigation is desirable to warrant its use in clinical practice.

#### Combination of autoantibodies

Growing evidences suggested that combining different autoantibodies are helpful for predicting treatment responses (Fig. 2b). For example, Julià et al. have demonstrated that the coexistence of RF and anti-CCP predicts a better response to TNFi, while having both anti-CarP and anti-PAD4 implies unresponsiveness [12]. On the other hand, combining anti-CCP and anti-MCV (anti-mutated citrullinated vimentin) antibodies can better identify RA patients who are more likely to have unfavorable TNFi responses [28]. Additionally, while anti-MMA alone is not associated with TNFi response, combining it with RF and anti-CCP leads to an increasing odds ratio for responders in a dose-dependent manner [24, 25]. Moel et al. reported that patients with a variety of antibodies against citrullinated, carbamylated, and acetylated peptides (ACPA, anti-CarP, AAPA) were found to have better responses to RA treatments [29]. These results corroborate earlier studies that a higher number of positive autoantibodies is correlated with a greater likelihood of positive treatment responses [25, 29–31]. This could be due to the increased inflammatory burden from the loss of self-tolerance to multiple autoantigens, rendering patients more susceptible to anti-inflammatory treatments.

#### Myeloid and lymphoid markers

Myeloid markers are serum proteins that originate from myeloid cells, and some of them have been found to be correlated with treatment responses, such as 14–3-3η and calprotectin. 14–3-3η are primarily intracellular chaperones overexpressed in synovial macrophages [32]. Since 14–3-3η are released into extracellular space upon TNF-α stimulation [33, 34], a lower serum level of 14–3-3η possibly indicates less involvement of TNF-α in the nature of disease. In such cases, therapeutic approaches alternative to TNFi may be more effective. As reported by Hirata et al., patients with lower 14–3-3η before treatment are more likely to achieve remission in treatment with tocilizumab [35].

Calprotectin is a heterodimer consisting of two small calcium-binding proteins S100A8 and S100A9. When calprotectin is released by neutrophils and macrophages in response to cell stress, it acts as danger-associated molecular patterns (DAMPs) to promote inflammation and joint destruction [36]. Upon our integrative survey, patients with elevated levels of calprotectin before treatments are more likely to have better treatment outcomes (Additional file 1: Fig. S1). Intriguingly, the interpretation of the relationship between calprotectin levels and treatment outcomes may be affected by the analytical methods. Studies using MS have reported a positive association between elevated levels of calprotectin and treatment responses to etanercept, whereas studies using immunoassays do not.

Lymphoid markers are serum proteins associated with lymphoid cells. C-X-C motif chemokine 13 (CXCL13), an important chemokine involved in the migration and development of B cell follicles within the synovium [37–40], is initially proposed to be a negative indicator of TNFi response [41]. Nevertheless, the pre-treatment levels of CXCL13 demonstrate conflicting associations with the clinical responsiveness to TNFi upon collective investigation [42] (Additional file 1: Fig. S1).

**Fig. 3:**
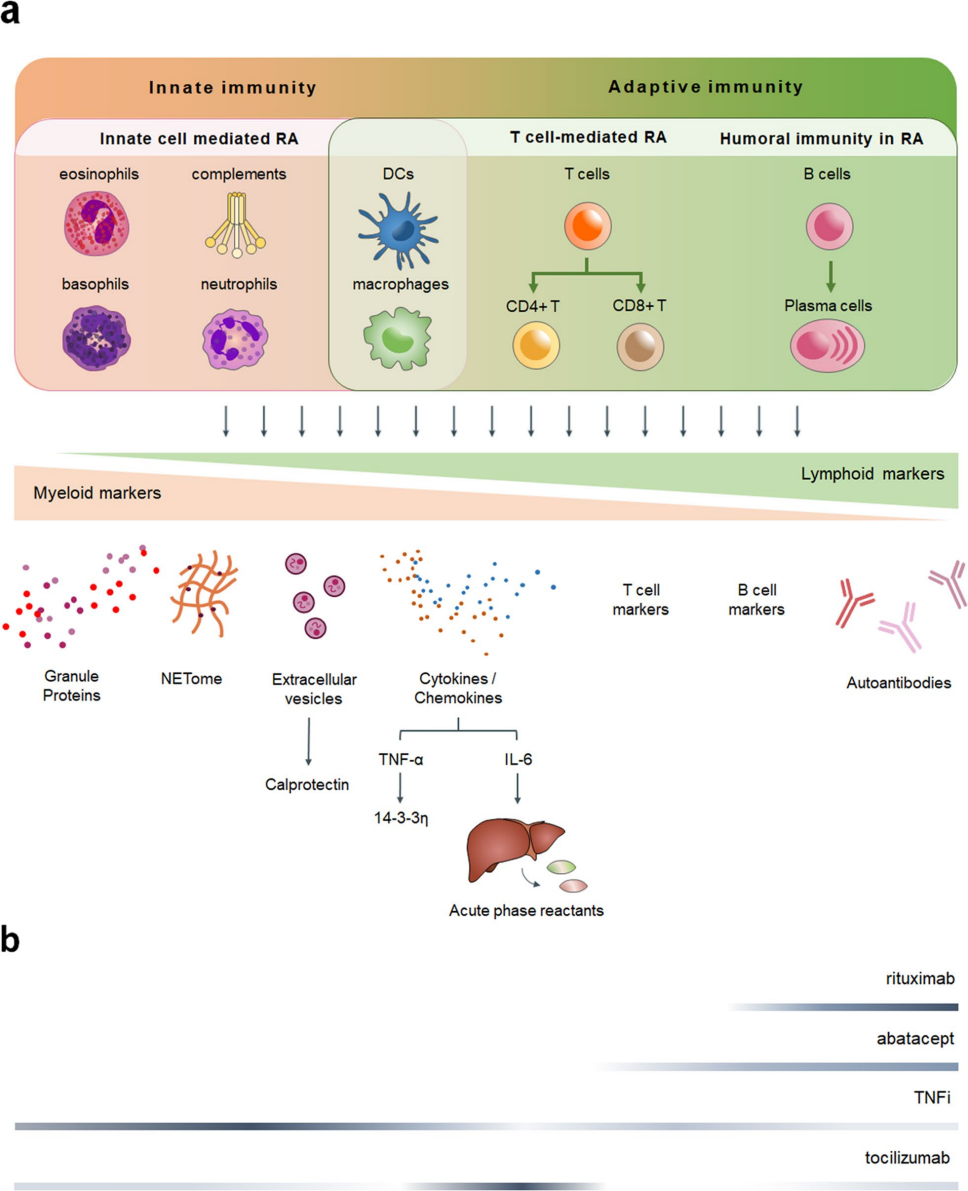
The immunopathology contributes to differences in serum proteome and the choice of treatments. **a** The spectrum of RA immunopathology ranges from innate immunity, cell-mediated to humoral immune response. The predominantly involved immune cells contribute to the distinctive compositions of serum proteins. DCs, dendritic cells; NETome, proteome associated with neutrophil extracellular traps. **b** Responders to four groups of bDMARDs are characterized by different biomarker spectrum, as illustrated by the color gradients. The darker areas of gradient lines represent enrichment of corresponding biomarkers, indicating the effective range of each bDMARD. The references are listed in Additional file 2: Table S3

The variation in immune cell populations has contributed to differences in the serum proteome (Fig. 3a) and is associated with the choice of therapeutic approaches (Fig. 3b). For example, RA patients with robust humoral immunity usually exhibit higher levels of autoantibodies, and rituximab and abatacept are recommended for these seropositive patients. In comparison, TNFi appears to be more effective for innate cell-mediated RA, especially for those enriched in myeloid markers, as proposed by Dennis et al. in which responders to TNFi were positively associated with synovial myeloid pathotypes [41]. Noteworthy, patients with multiple antibodies positive also benefit from TNFi, suggesting that TNFi may be effective across a wide range of both cell-mediated and humoral immune responses. The four groups of bDMARDs could be complementary with each other, and alternative treatments targeting different immunopathologies are suggested for inadequate responders to TNFi.

### Early evaluation of therapeutic effectiveness for treatment adjustments

**Fig. 4:**
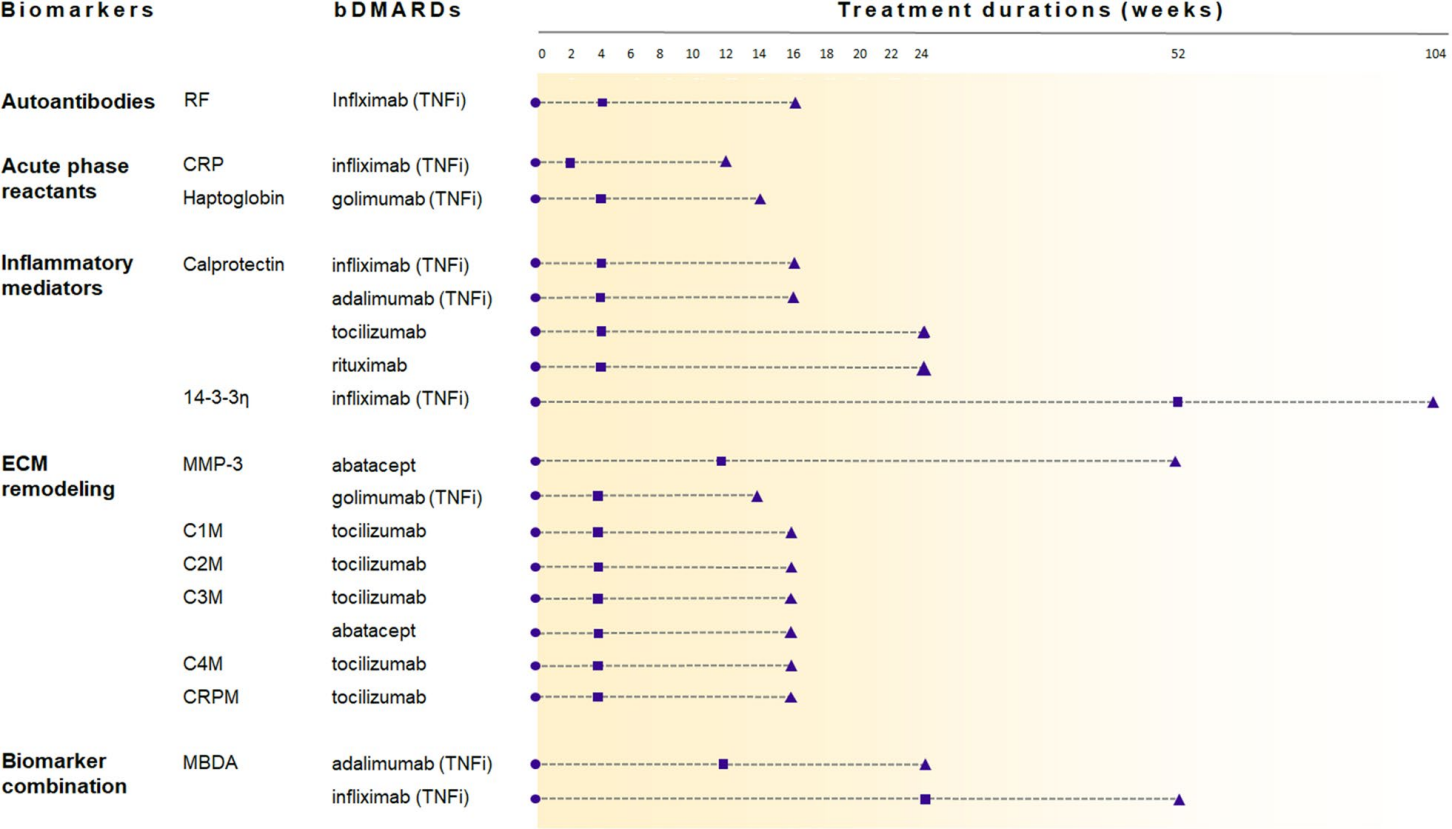
The concentration changes of serum proteins in the prediction of treatment responses. Biomarker concentrations were measured at treatment initiation (circles) and the early stage (squares), while clinical responses were evaluated at the end of treatment (triangles). The decrease in the levels of the listed biomarkers was associated with positive responses at the end of treatment. The references are provided in Additional file 2: Table S4, and the full names of biomarkers are listed in Additional file 2: Table S5. *ECM* extracellular matrix

Early changes in the concentrations of serum proteins can also serve as predictors of treatment outcomes. For example, a prominent decrease in C-reactive protein (CRP) within the first two weeks of treatment is associated with a favorable outcome at week 12 [43, 44]. A reduction in haptoglobin and other acute phase reactants by week 4 is also linked to better responses at week 14 [45]. In addition, similar observations have been noticed in several serum proteins, including autoantibodies, inflammatory mediators and extracellular matrix (ECM) markers. The relationship between the dynamics of these serum proteins and the prediction of subsequent treatment responses has been reported in treatments with TNFi (infliximab, adalimumab, golimumab), tocilizumab, abatacept and rituximab, as summarized in Fig. 4.

#### Autoantibodies

The extent to which RF levels decrease serves as an early indicator of therapeutic effectiveness in several TNFi (infliximab and adalimumab) and tocilizumab [46–49]. Patients with decreasing RF levels during treatments ultimately have better treatment outcomes (Fig. 4). However, the degree of autoantibodies changes is unable to predict clinical improvement in rituximab [88–91].

The glycosylation profile of autoantibodies offers an additional insight. The effector functions of antibodies are modulated by the glycan structures on the Fc portion of IgG. The IgG glycosylation profile varies in different pathological conditions. It has been proposed that effective RA treatments would lead to a significantly increase of glycosylation [50–54], and patients with more increases in IgG galactosylation at the initial stage are more likely to have clinical improvement at the final assessment [53, 54]. Meanwhile, as reported by Ciregia et al., patients attaining clinical responses exhibit higher degrees of glycosylation in corticosteroid-binding globulin and lipopolysaccharide-binding protein after 12 months of treatments with corticoids, methotrexate, and bDMARDs [55]. Further research on the glycosylation profiles of serum proteins may be important in discovering predictors of RA treatment responses.

#### Inflammatory mediators

The dysregulation of immunological pathways in RA leads to the aberrant activation of inflammatory mediators, followed by erosion of cartilage and bone in joints [36]. Calprotectin has been regarded as a potential biomarker as the serum and synovial fluid levels of calprotectin are significantly increased in RA patients. Studies have demonstrated that patients with remarkable decreases in calprotectin levels after treatments with bDMARDs are more likely to experience favorable responses [56–58]. Similar observation is also found in 14–3-3η upon treatments with TNFi, tocilizumab and tofacitinib [32, 35, 59–61].

In contrast to calprotectin and 14–3-3η, other therapeutic targets, such as TNF-α and IL-6, are barely modulated by their corresponding inhibitors [54, 62–64]. The fast-acting and short-lived nature of cytokines make it difficult for accurate quantification. To address the challenge, Schotte et al. [65] and Dissanayake et al. [66] have utilized the enzyme-linked immunosorbent spot assay (ELISpot) to assess cytokines secreted by peripheral blood mononuclear cells. Still, cytokines do not serve as an ideal indicator for the treatment responses.

#### Extracellular matrix (ECM) remodelling

While it remains challenging to characterize cytokines, the downstream products such as the components of ECM are relatively long-lasting and directly reflect structural changes in joints. There are three categories of ECM markers, including bone remodelling, cartilage modulation, and synovial inflammation. Most bDMARDs demonstrated a positive effect on bone remodelling, with varying degrees of reductions in bone resorption markers and increments in bone formation markers [67–69]. Patients with early reduction in bone resorption markers, such as the decrease of receptor activator of nuclear factor kappa-B ligand, are more likely to experience favorable outcomes after six months of treatments with abatacept [70, 71]. As opposed to bone remodelling, most bDMARDs exert modest effects on cartilage modulation [72–74].

Matrix metalloproteinases (MMPs) are crucial mediators in proteolytic degradation and synovial destruction. A rapid downregulation of matrix metalloproteinases 3 (MMP-3) is found to be an early indicator of a favorable outcome to TNFi, tocilizumab, sarilumab and abatacept [17, 44, 68, 75], suggesting that successful suppression of MMP-3 activity at the beginning of treatment is able to impede progressive joint damage. On the other hand, there are several MMP-mediated products resulting from the degradation of type I collagen (C1M), type II collagen (C2M), type III collagen (C3M), type IV collagen (C4M) and CRP (CRPM). Decreases in the levels of these products indicate the reversal of ongoing synovial damage and better clinical responses [44, 75–78]. The extent of reduction in these markers within the first four weeks of tocilizumab treatments can aid in early distinguishing between responders and non-responders [77, 79, 80], and a similar phenomenon is also found in baricitinib [81]. While ECM markers were less useful for predicting therapeutic responses before treatment, the robustness of these findings suggested that the reduction in ECM markers may precede clinical outcome and enable early prediction of treatment results.

#### Combination of biomarkers

Considering the heterogeneous nature of RA, utilizing multiple biomarkers for prediction is an alternative solution. The multi-biomarker disease activity (MBDA) score, which is based on the measurement of 12 serum proteins (cytokines, growth factors, ECM and stress-related proteins), has been recognized as an objective assessment of disease activity and a predictor of radiographic progression [82–85]. Moreover, early changes in MBDA scores have been reported to be associated with clinical remission in treatments with TNFi (infliximab, adalimumab, and etanercept), rituximab, and tofacitinib [73–77].

In an era of high-throughput technology and advanced computational analysis, combining various molecular signatures to characterize clinical response has become a popular strategy. For instance, early changes in immunophenotyping and synovial cellular population enable the prediction of treatment responses [86, 87]. Tasaki et al. have developed statistical models to calculate the probability of patients classified as RA from the aspects of transcripts, proteins, and immunophenotypes [6]. They noticed that the difference in reduction of RA probability between responders and inadequate responders is more prominent based on a proteomic model than a transcriptional model, implying that serum proteome provides a stronger evidence towards healthy states. Furthermore, a greater reduction in the calculated probability after the first month of treatment is associated with a higher likelihood of clinical responsiveness at the 24^th^ week. The early alteration in molecular signatures facilitates the prediction of subsequent responses, enabling a timely decision for clinical treatments.

### Therapeutic drug monitoring during treatment

The variability of drug concentrations in serum is a critical issue for precision medicine. Therapeutic drug monitoring (TDM, monitoring the drug concentrations during treatments) provides a clue for the optimization of personalized treatments. Additionally, the production of anti-drug antibodies (ADAs) upon exposure to exogenous therapeutic proteins emerges as another important consideration. ADAs partly diminish the therapeutic effects of patients’ medications and the measurement of ADAs is potentially helpful in determining the cause of suboptimal drug level and aids in treatment modifications. The application of TDM in the management of RA has received growing attention (Table 1).

**Table 1:** Therapeutic drug monitoring in the prediction of rheumatoid arthritis treatment

bDMARDs	TNFi			IL-6 receptor inhibitors		CTLA4–Ig	Anti-CD20
	Adalimumab	Infliximab	Etanercept	Tocilizumab	Sarilumab	Abatacept	Rituximab
Association between the drug levels and the prediction of clinical results							
Clinical responses	▲ [89, 90]	▲ [88, 92, 93]	▲ [94]	▲ [91, 95]			
Successful dose reduction	ʘ [96–98]	ʘ [99]	ʘ [97, 98]	ʘ [97]			
Association between the positivity of anti-drug antibodies (ADAs) and the prediction of clinical results							
Drug levels	▼ [105, 106]	▼ [102, 103, 106, 108–112]	ADAs are mostly undetectable in etanercept	▼ [107, 113]	▼ [107, 113]		
Clinical responses	▼ [89, 90, 102, 103, 105, 108, 109, 114–116]	▼ [92, 102, 103, 115]		ʘ [118, 119]	ʘ [107, 113, 118, 120]	ʘ [102]	ʘ [102, 121]
Successful drug switching	▲ [124] ʘ [125]	▲ [124] ▼ [149]					

▲ Suggests that patients with higher drug levels or positive ADAs are more likely to experience clinical improvements in corresponding scenarios, while ▼ indicates otherwise

ʘ Represents that drug levels or ADAs may not be useful for therapeutic decisions in corresponding scenarios

#### Measurement of drug levels

The drug concentrations directly reflect the effectors of clinical responsiveness. Patients with higher drug levels are expected to experience clinical improvement. Studies have shown that the drug levels of infliximab, adalimumab, etanercept, and tocilizumab are positively associated with their clinical responses [88–95]. Nevertheless, it is not encouraged to reduce the dose of infliximab, adalimumab, and etanercept based on serum drug concentrations given current evidences [96–99].

#### Measurement of anti-drug antibodies

The formation of anti-drug antibodies (ADAs) is a major reason for inter-individual variations in serum drug levels [100, 101]. The prevalence of ADAs ranges from nearly undetectable in etanercept to 67% in infliximab and adalimumab [102, 103]. Factors influencing the emergence of ADAs include genetic predisposition, smoking habits, manufacturing process of bDMARDs, and the prolonged exposure of bDMARDs, in which the last factor is the riskiest [104]. Serum drug levels are affected by ADAs through various biological mechanisms, such as competitive inhibition or enhanced drug clearance. Most studies agree that ADAs against infliximab and adalimumab negatively impact the drug level [102, 103, 105–113], thereby hampering the accomplishment of therapeutic responses [89, 90, 102, 103, 105, 108, 109, 114–117]. Conversely, ADAs against tocilizumab, sarilumab, and rituximab are less relevant to clinical responses [102, 107, 113, 118–121]. Ongoing monitoring is still necessary to establish the clinical relevance, especially for patients with a higher risk of immunogenicity [122].

For patients with inadequate improvement under a given treatment, switching to another class of bDMARDs may be a more effective strategy than dose escalation [123]. Jamnitski et al. proposed that patients who developed ADAs to infliximab or adalimumab are more likely to have favorable responses after switching to etanercept [124]. Nevertheless, the use of ADAs for drug switching remains controversial [125].

### Predicting successful treatment withdrawal

When the clinical symptoms of RA are well-controlled, physicians may start to explore the possibility of treatment de-escalation. Although treatment tapering has been recommended in recent guidelines [1], scarcely had it been implemented in clinical practice under the concern of disease flares [126, 127]. It would be of great help if one could identify patients most likely to benefit from treatment withdrawal.

Common clinical variables (e.g. age and swollen joint count) have been extensively discussed, but they are not recommended as ideal predictors [128, 129]. Molecular remission, on the other hand, has drawn an increasing attention because it requires a more stringent criterion that patients’ molecular profiles need to be close to those of healthy individuals. Identifying the serum proteome associated with the molecular remission has become a fascinating field. Table 2 summarizes the patient cohorts, interventions, primary outcomes, and clinical implications of recent efforts, in which nine studies agreed that seropositive patients have higher rates of disease relapses during tapering of tocilizumab and TNFi [29, 130–137]. In particular, the risk of relapses increases as more antibody reactivities are involved [29, 134]. Patients positive for a broad spectrum of autoantibodies are not only more responsive to bDMARDs, but also more likely to experience disease relapses during treatment discontinuation, highlighting the need of continuous treatments for these seropositive patients.

**Table 2:** Potential biomarkers associated with successful treatment withdrawal in rheumatoid arthritis

Cohorts	Previous treatments	Inclusion criteria (definition of remission)	Interventions	No. of patients	Primary outcomes	Clinical implications	Refs.
Leiden EAC and ERAS	csDMARDs	defined by rheumatologist	Stop all csDMARDs	1349	Sustained DFR > 1y until last follow up	RF ▼ anti-CCP ▼ Patients positive for RF and/or anti-CCP were less likely to reach sustained DFR	[119]
Spain	bDMARDs for 2 m	Boolean definition at least 12 m	Taper bDMARDs	77	Taper failure in 40 m	RF ▼ anti-CCP ʘ Patients positive for RF were associated with taper failure, but the association was not observed in anti-CCP	[120]
RETRO	TNFi or tocilizumab > 6 m	DAS28-ESR < 2.6 at least 6 m	Taper or stop TNFi and tocilizumab	101	Flare: DAS28-ESR ≥ 2.6 in 12 m	anti-CCP ▼ anti-CCP-positive patients were more likely to relapse	[121]
				94	Flare: DAS28-ESR > 2.6 in 12 m	anti-CCP ▼ MBDA ▼ Patients with lower MBDA score (< 30 units) and negative anti-CCP are at higher risk of relapse after treatment withdrawal	[122]
				94	Flare: DAS28-ESR > 2.6 in 12 m	Diverse autoantibodies ▼ The risk of relapse increased with more autoantibody reactivities, regardless of specific isotypes or targets	[123]
				57	Flare: DAS28-ESR > 2.6 in 12 m	Calprotectin ▼ Patients with higher calprotectin levels at the moment of treatment withdrawal were prone to relapse	[127]
IMPROVED	csDMARDs (methotrexate and prednisolone) for 4 m	DAS44 < 1.6	Taper and stop methotrexate at 8 m	399	Sustained DFR between the 1st–2nd year of follow up	Diverse autoantibodies ▼ Patients with more diverse autoantibodies were less likely to achieve DFR	[19]
				610	Sustained DFR > 1y until last follow up (2y)	RF ▼ anti-CCP ▼ RF and/or anti-CCP positive patients were less likely to reach DFR	[124]
				104	Flare: DAS44 ≥ 1.6 in 12 m	Calprotectin ▼ Patients with higher calprotectin levels at the moment of treatment withdrawal were prone to relapse	[127]
RRRR trial	infliximab (TNFi) for 1y	SDAI ≤ 3.3	Stop infliximab	337	Sustained DFR > 1y until last follow up (2y)	RF ▼ TNF-α ▲ Patients with lower RF and higher TNF-α have a higher likelihood of sustained DFR	[125]
BioRRA	csDMARDs alone, no prior bDMARDs	DAS28-CRP < 2.4	Stop all csDMARDs without tapering	44	Flare: DAS28-CRP ≥ 2.4 in 6 m	39 serum proteins; RF ▲ anti-CCP ʘ IL-27 ▼ RF status, two cytokines/chemokines (IL-27, MCP-1), and three CD4 + T cell genes were associated with the risk of flare after DMARD cessation. Patients with lower IL-27 were more likely to remain remission	[126]
POET	TNFi > 1y	DAS28-ESR < 3.2 at least 6 m	Stop TNFi	439	Flare: ΔDAS > 0.6 and DAS28-ESR ≥ 3.2 in 12 m	MBDA ▼ Patients with higher MBDA score at the moment of treatment withdrawal were more likely to relapse	[129]
STRASS	TNFi	sustained DAS28 remission	Taper TNFi	137	Flare: ΔDAS > 0.6 and DAS28 > 2.6 in 18 m	MBDA ʘ No significant difference in MBDA scores between relapsing and non-relapsing patients	[130]
DRESS	stable adalimumab or etanercept (TNFi) > 6 m	DAS28-ESR < 3.2 at least 6 m	Taper and strop adalimumab and etanercept	115	Flare: ΔDAS > 0.6 and DAS28 ≥ 3.2 OR ΔDAS > 1.2 in 18 m	MBDA ʘ The MBDA scores at the moment of tapering were not associated with the risk of relapse after TNFi withdrawal	[131]
SURPRISE	tocilizumab ± methotrexate for 1y	DAS28 < 2.6	Stop tocilizumab, keep methotrexate	105	Sustained DFR > 1y	RF ▼ MMP-3 ▼ Patients with negative RF and lower MMP-3 were more likely to achieve DFR	[139]
FLAIR	bDMARDs for 2y	SDAI ≤ 3.3 at least 3 m	Stop bDMARDs	36	Flare: ∆DAS28-ESR > 0.6 and DAS28-ESR ≥ 3.2 in 24 m	12 cytokines: soluble TNFR1 ▼ IL-2 ▲ Patients with lower levels of soluble TNFR1 and higher levels of IL-2 were more likely to remain remission	[140]
UMIN 000044434	TNFi or tocilizumab	DAS28-CRP < 2.3 at least 1y	Stop TNFi and tocilizumab, keep other medication	40	Flare: DAS28-CRP ≥ 2.3 in 24 m	73-plex cytokine array: IL-34 ▼ IL-19 ▲ Upon treatment withdrawal, the IL-34 was significantly up-regulated and IL-19 was significantly down-regulated in patients experiencing subsequent relapses	[141]
U-Act-Early	tocilizumab	DAS28 < 2.6 at least 24 wk	Taper and stop tocilizumab	24	Sustained DFR ≥ 3 m until last follow up (2y)	85 inflammatory proteins (Luminex multiplex assay): 14 proteins corresponding to leukocyte activation pathway are associated with sustained DFR	[142]

*bDMARDs* biological DMARDs, *csDMARDs* conventional synthetic DMARDs, *DFR* DMARD-free remission, i.e., fulfilling the definition of remission after DMARD cessation; *TNFi* TNF inhibitors; *SDAI* Simple Disease Activity Index, *MBDA* multi-biomarker disease activity, *MCP-1* monocyte chemoattractant protein-1, *TNFR1* tumor necrosis factor receptor 1, *wk* week(s), *m* month(s), *y* year(s)

▲ Suggests that elevated levels of designated biomarkers are associated with higher rates of successful treatment withdrawal (favorable)

▼ Indicates that elevated levels of designated biomarkers are associated with higher rates of relapse (unfavorable)

ʘ Implies that the levels of designated biomarkers are less relevant to the success rates of treatment withdrawal

In our literature survey, two studies have demonstrated that patients with higher calprotectin levels at the moment of dose reduction were predisposed to relapse [138, 139], and four studies have investigated the application of MBDA in making treatment adjustments [133, 140–142]. Higher MBDA scores at the moment of intervention were positively associated with the risk of flares after treatment tapering [133] or cessation [140], but similar observations were not reported in other cohorts [141, 142]. More importantly, patients with a low MBDA score (< 30 units) and negative anti-CCP are at a lower risk of relapses, suggesting that the combination of MBDA score and anti-CCP status enables risk stratification for treatment withdrawal [143].

Generally speaking, lower levels of autoantibodies, calprotectin, and MBDA are associated with reduced risks of relapses. Persistent elevations of these serum proteins are implicated in subclinical inflammation and may hinder successful treatment de-escalation. The state in which the levels of molecules are akin to healthy controls is considered a more stabilized condition for tapering of bDMARDs. Patients attaining molecular remission are more appropriate for treatment adjustment as compared with those only having clinical remission, and the probability of sustained disease inactivation increases when more molecular classes achieve remission [6, 144]. A recent study reported by Inamo et al. has elucidated a distinct subset of CD4 + and CD8 + as the key components associated with molecular remission [7]. With multi-omics approaches, Tasaki et al. concluded that downregulation of neutrophils (and upregulation of natural killer cells) are correlated to remission in transcript-based models, whereas inactivation of the complement pathway is associated with remission in protein-based models [6, 145].

## Conclusions and future directions

Personalized medicine remains an unmet need for RA patients. The considerable heterogeneity at the proteomic level has contributed to variations in therapeutic responses. In this review, we comprehensively summarize the clinical applications of serum proteins in different treatment stages. Upon collective investigation, some serum proteins that were initially suggested to be promising predictors in individual studies were found to have inconsistent associations. Our survey suggests that autoantibodies, calprotectin and 14–3-3η have a more consistent potential in predicting therapeutic outcomes, giving directions for further validation. We also shed light on the spectrum of RA immunopathology underlying between responders to different treatments. RA patients who exhibit dominant humoral immunity are more likely to respond to rituximab and abatacept, but they are also prone to relapse upon treatment cessation. On the other hand, RA patients with predominant cell-mediated immunity and myeloid cells tend to respond to TNFi. Serum protein profiling reveals a novel insight for personalized treatment strategies.

MS and immunoassays are currently the major analytical platforms for proteomic analyses. In our survey, complement components and apolipoproteins are commonly reported in the articles using MS, while cytokines, chemokines, and 14–3-3η proteins are frequently discussed in the studies using immunoassays (the analytical strategies of these articles are listed in Additional file 2: Table S3). Our observation is consistent with the article reported by Skalnikova et al. that shotgun proteomics might have difficulties in the detection of low abundant proteins (such as cytokines) without enrichment [3]. Another possible reason is the low molecular weights of cytokines, (e.g., the protein sequence lengths of IL-6 and TNF-α are 212 and 233, respectively) further pose challenges for their detection by shotgun proteomics. It is noteworthy that calprotectin can be identified using immunoassay-based and MS-based proteomics, yet the statistical significance varies between the two platforms, which highlights the need for a cross-platform normalization tool [146].

While current studies have identified several serum protein candidates, their validation in large clinical cohorts is essential for clinical practice. The challenges of biomarker discovery include the heterogeneous size and clinical features of patient cohorts, variability in analytical platforms, and the lack of reproducibility. As incorrect prediction may pose additional side effects and costs to the patients, ongoing researches are dedicated to combining different biomarkers using omics approaches, and refining proteomic techniques to address these issues. More advanced proteomic techniques, such as single-cell proteomics and imaging MS, are expected to improve the identification of protein biomarkers with higher sensitivity and specificity [147]. Recently, the incorporation of serum proteins as prediction models into the clinical care of RA has drawn increasing attention because it not only facilitates clinical decision-making, maximizes the possibility of successful treatments, but also reduces costs [5, 148]. In summary, our study provides a comprehensive discussion of the relationship between serum proteins and RA treatments, hoping to pave the way for precision medicine and overcome the limitation of the traditional "one-size-fits-all" approach for RA patients.

## Supplementary Information

**Additional file 1**. A pdf file including Fig. S1. Association between the pretreatment levels of serum proteins and clinical responses of RA treatments.

**Additional file 2.** An excel file including Table S1. Search strategy. Table S2. Studies associated with autoantibodies (References of Fig. 2). Table S3. Studies associated with serum proteins other than autoantibodies (References of Fig. 3b and Fig. S1). Table S4. References of Fig. 4. Table S5. Serum proteins and their full names.

## Data Availability

Data are available from the corresponding author upon reasonable request.

## Abbreviations

ACPAs: Anti-citrullinated protein antibodies
ADAs: Anti-drug antibodies
Anti-CarP: Anti–carbamylated protein
Anti‐CCP: Antibodies against cyclic citrullinated peptide
bDMARDs: Biological disease-modifying anti-rheumatic drugs
DFR: DMARD-free remission
ECM: Extracellular matrix
IL: Interleukin
MAA: Malondialdehyde-acetaldehyde adducts
MBDA: Multi-biomarker disease activity
MMPs: Matrix metalloproteinases
RA: Rheumatoid arthritis
RF: Rheumatoid factors
TDM: Therapeutic drug monitoring
TNFi: Tumor necrosis factor inhibitors
