# Supplementary material for: Tailored therapeutic decision of rheumatoid arthritis using proteomic strategies: how to start and when to stop?
Source: Clin Proteomics. 2023 Jun 10;20:22. doi: 10.1186/s12014-023-09411-2 (PMC10257292; doi:10.1186/s12014-023-09411-2)
Supplement: Supplementary file 1 — Additional file 1. A pdf file including Fig. S1. Association between the pretreatment levels of serum proteins and clinical responses of RA treatments. [file 12014_2023_9411_MOESM1_ESM.pdf]

Additional file 1 for

Tailored therapeutic decision of rheumatoid arthritis using proteomic strategies: How to start and when to stop?

Shuo-Fu Chen, Fu-Chiang Yeh, Ching-Yun Chen, Hui-Yin Chang

Contents

Additional file 1: Fig. S1 Association between the pretreatment levels of serum proteins and clinical responses of RA treatments

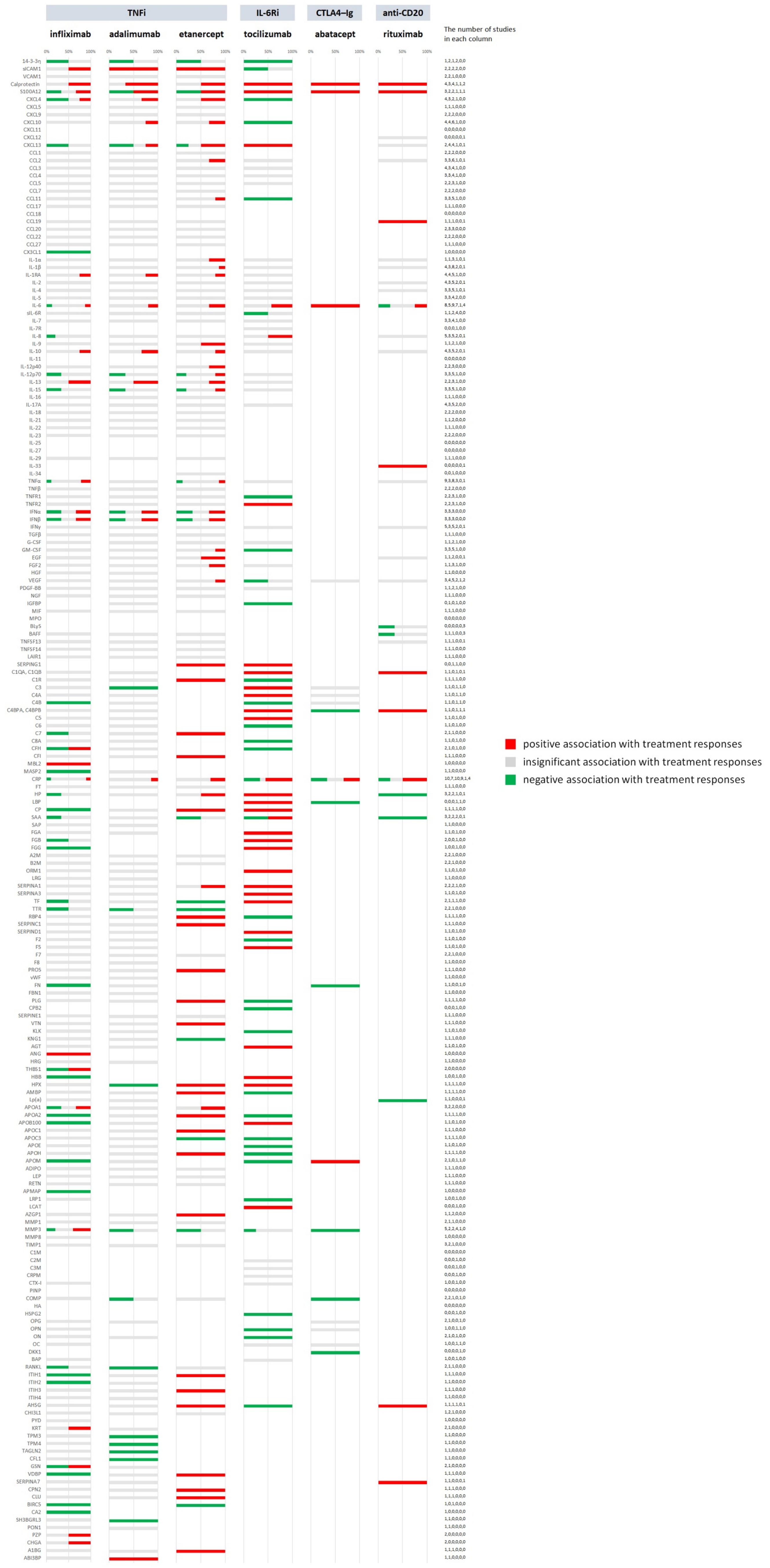

Additional file 1: Fig. S1 Association between the pretreatment levels of serum proteins and clinical response of RA treatments

The percentages of included studies supporting positive, negative, and insignificant associations with the treatment responses are displayed in red, green, and grey bars, respectively. The number of included articles for each column is listed on the rightmost column. The references are provided in **Additional file 2: Table** S3. The full names of serum proteins are listed in **Additional file 2: Table S5**.
